# Epidemiology and financial loss estimation of blackleg on smallholder cattle herders in Kembata Tambaro zone, Southern Ethiopia

**DOI:** 10.1186/s40064-016-3541-2

**Published:** 2016-10-21

**Authors:** Birhanu Ayele, Worku Tigre, Benti Deressa

**Affiliations:** 1 Faculty of Veterinary Medicine, University of Gondar, P.O. Box 196, Gondar, Ethiopia; 2 School of Veterinary Medicine, College of Agriculture and Veterinary Medicine, Jimma University, P.O. Box 307, Jimma, Ethiopia

**Keywords:** Blackleg, Cattle, Cumulative incidence, Financial benefit, Financial cost, Southern Ethiopia

## Abstract

**Background:**

Blackleg is one of the major bacterial infections causing tremendous economic losses to cattle herders in many parts of Ethiopia. Despite the huge burden, no comprehensive studies have quantified the impact or its distribution throughout the country. This study was aimed to estimate the epidemiological aspect of blackleg, financial costs and benefits of its control through annual vaccination on small holder cattle herders in Southern Ethiopia.

**Methods:**

Annual financial cost due to blackleg was calculated as the sum of production losses due to mortality, morbidity, treatment and vaccination costs at herd level. Production loss due to the disease was estimated and compared between local zebu and crossbred cattle. Partial budget analysis was used to estimate financial benefit of control intervention through annual vaccination.

**Results:**

An overall cumulative incidence and mortality rate of blackleg in local zebu cattle population was 17.9 % (95 % CI 16.5–19.4) and 3.6 % (95 % CI 2.9–4.4 %) respectively. Cumulative incidence and mortality rate attributed to blackleg in crossbreds cattle was 19 % (95 % CI 16.9–21.6) and 3.9 % (95 % CI 2.9–5.3 %) respectively. There was no statistically significant difference (p > 0.05) in both variables between the two breeds. Financial costs in blackleg infected herds was estimated to be USD 9.8 (95 % CI 6.7–14.4) per head for local zebu and USD 16 (95 % CI 10–24.4) per head for crossbred cattle. The marginal rate of return that could be obtained from the control intervention was estimated to be 9 (900 %) and the net benefit per head was USD 0.4 for local zebu and USD 0.8 for crossbred cattle. Vaccination, therefore, reduces financial losses due to blackleg by 3.4 and 6.9 % per head in local zebu and crossbred cattle herds respectively.

**Conclusions:**

The present study revealed considerable financial losses due to blackleg occurrence. The information obtained would be helpful to improve the farmers’ livelihood and may open new avenues of research for the eradication and control of the disease at local and national level.

## Background

Ethiopia is believed to have the largest livestock population in Africa (CSA 2013; Solomon et al. 2003; Tilahun and Schmidt 2012) contributing 16.5 % of the national Gross Domestic Product (GDP) and 35.6 % agricultural GDP (Metaferia et al. 2011). Livestock also serve as means of livelihood for lots of rural community in Ethiopia. Farmers generate household income through sale of animals or sale and consumption of animal products. Livestock are form of savings and insurance for the poor as sale of animals provide immediate cash to deal with significant or unexpected expenditures (as school or medical fees); livestock provide social and cultural contributions like, gifts for friends and relatives, inheritance, marriage (bride wealth, dowry), compensation, making peace after conflict and etc (Behnke 2010).

Despite the enormous contribution, productivity is constrained by several factors from which disease being the most important (Solomon et al. 2003; Benin et al. 2003; Jabbar et al. 2007; Negassa et al. 2011). It is estimated that the aggregate annual economic loss from animal diseases through direct mortality and reduced productive and reproductive performance in Ethiopia is US$ 150 million (Admasu 2002).

Blackleg is one of the major bacterial infections causing tremendous economic losses to cattle herders in many parts of the country (Rashid and Shank 1994). The economic losses caused by the disease are mainly due to deaths in affected animals. Losses in milk production and reduction in working capacity of farm animals are the additional costs the disease wields on affected animals. The causative agent, *Clostridium chauvoei*, is Gram-positive, motile, rod-shaped anaerobic bacterium that can produce environmentally persistent spores when conditions are not ideal for growth (Sarah 2013). The spores can remain in the soil for years in an inactive state, and return to their infectious form when consumed by grazing livestock (Merck 2005; Sarah 2013; Radostits et al. 1995).

Most losses due to blackleg occur when cattle are between the ages of 6 months and 2 years (Merck 2005; Sarah 2013). Typically, cattle that have a high feed intake and are well conditioned tend to be most susceptible. Lesions develop without any history of wounds, although bruising or excessive exercise may precipitate disease in some cases (Merck 2005). Blackleg seldom affects cattle older than 2 years of age, most likely due to immunity induced by vaccines or natural exposure. However, sporadic cases do occur in cattle older than 2 years and are often associated with the reuse of needles for multiple injections. It is also indicated blackleg to be a problem in cattle less than 4 months old that do not receive adequate passive immunity through colostrums (Alabama 2013).

Ethiopian Ministry of Agriculture reported 2553 blackleg outbreaks and a total of 5134 deaths during 2007/08–2009/10 years from different districts of the country (MoA 2010). However, the figure reported during the period is much smaller than the actual number of outbreaks and deaths as a number of zones with large number of cases did not report in the same years. The overall mortality rate of blackleg as indicated by the report in Ethiopia was by far smaller when compared with the mortality findings of the disease in veterinary literatures, as case fatality rate of blackleg (*Clostridium chauvoei*) in fully susceptible populations approaches 100 % (Merck 2005; Radostits et al. 2006). The less mortality rates might be due to the endemic nature of the disease in Ethiopia in that animals in endemic area might obtain certain protective active immunity from bacterial infections that enables them to conquer effect of the disease. Besides, less virulent form of blackleg might existence in Ethiopia that was not seen in other areas of the world. However, further study would be necessary to determine the underpinning reasons.

Regarding blackleg treatment, farmers use modern (veterinary) drugs alongside traditional (herbal) remedies immediately after diagnosing the cases. Antibiotics like Procaine penicillin and Oxytetracyclines are commonly used in areas where blackleg is major concern, and treatment regime may extend from 3 to 5 days depending on severity of the disease. Control relies mainly on vaccination carried out at the onset of an outbreak using the whole broth culture suspension of *Clostridium chauvoei* vaccine (local isolate) produced by the National Veterinary Institute (NVI, Ethiopia).

Kembata Tambaro is one of the Ethiopian zones where blackleg is endemically established and known for severely affecting herders’ livelihood according to the zone’s livestock disease reporting manual. Recent study conducted in the area to identify the major livestock diseases also showed blackleg as one of the priority cattle diseases (Ayele et al. 2015). Despite the huge burden no comprehensive studies have quantified the impact or distribution of the disease in the area where cattle are important assets to the local farmers. Therefore, the objectives of this study were to investigate the epidemiological aspect of blackleg on smallholder cattle herders in Kembata Tambaro zone, and also to estimate the financial cost and benefits of its control at household level through annual vaccination.

## Methodology

### Description of the study area

This study was conducted in Kembata Tambaro zone; Southern Nations, Nationalities and Peoples Region of Ethiopia. The zone possesses 7 woredas, 120 rural and 22 urban kebeles (smallest administrative unit in Ethiopia) and 14, 1952 hectares of land. It has a total human population of 828,002. About 14.3 % of the populations live in urban while the remaining 85.7 % are rural dwellers (ARDO 2011).

Kembata Tambaro zone is gifted with enormous livestock resource and livestock contribute to household livelihoods through a variety of direct and indirect ways. The estimated livestock population of the zone is 169,265 cattle (composed of 140,432 local zebu and 28,833 crossbred), 250,736 sheep and goats, 61,133 equine, 339,712 poultry and 34,095 bees. Cattle play essential role to the farming economy. A draft oxen power is used for the production of agricultural crops, milk and milk product is used for household consumption and sale and animal manure for fertilizer. Many of the farmers usually generate income by selling milk and milk products, fattening mature male animals, and barren and culled females (ARDO 2011).

### Study design

Cross-sectional study using participatory appraisal technique (focus group discussion) and questionnaire survey were carried out from November 2012 to March 2013.

### Sampling technique and sample size determination

Four districts were selected purposively for this study. Selections of districts were made in consultation with zonal livestock health experts based on blackleg occurrence records from the year preceding the start of the study. Eighteen (18) peasant associations (PAs) were randomly included from the selected districts. Households were the final sampling units.

A total of 528 respondents participated in the study, 198 focus group discussants and 330 respondents of house-to-house interview. To increase the accuracy of information generated, households recognized by the community as knowledgeable in cattle rearing and indigenous veterinary knowledge were purposively included as key members of the focus groups (Ayele et al. 2015).

Sample size for respondents of house-to-house interview was determined using the formula (n = 0.25/SE2) given by Arsham (2002) at the standard error (SE) of 0.027 with 95 % confidence level. Initially, sampling frame was constructed from a list of all households in each village. A recruitment exercise was done to identify households who have a herd of cattle and willing to participate in the study. Then, random selection was used to sample volunteer households who had not participated in focus group discussion. Herd, for the purpose of this study was defined as a collection of different breed, age and sex of cattle that are owned by a single farmer or household. Thus, in the context of this study, a herd is equivalent to a household.

### Study methodology

#### Participatory appraisal method

Financial impact of blackleg on milk yield and draft power output in the study districts was estimated based on participatory appraisal methods (focus group discussions). One focus group having 6–12 members was organized in every of the selected PAs as shown in Ayele et al. (2015). Milk losses due to blackleg were quantified first by questioning the discussants to estimate the normal parameters that healthy animals produce in their localities. For instance, discussants were asked to explain the average daily milk production of local zebu and cross breed healthy cows using the container that they used to collect milk. The local container was then filled with water to the mark specified by the discussant and the absolute volume of water was measured with a standard liters graduated container. Discussants were asked to show the milk produced by cows suffering from blackleg by marking the volume on the local container. Again the absolute volume was measured with a graduated container and recorded to calculate the difference. Discussants were asked to specify the duration (number of days) blackleg could have its effect on milk production and draft power. All the data were recorded for subsequent calculation of production loss attributed to blackleg.

#### Questionnaire survey

Epidemiological parameters related to blackleg occurrence and associated financial losses from herders’ perspective were assessed using the questionnaire surveys. The questionnaires were prepared based on veterinary literatures and in consultation with some local peoples. One animal health assistants and a traditional healer were interviewed regarding the local name of blackleg in each of the selected PA. Farmers’ ability to identify blackleg infection was cross-checked by enquiring about the clinical signs of the disease. Each farmer who reported that blackleg had infected his or her herd was asked to describe the clinical signs of the disease. For those who mentioned clinical signs shared by the diseases easily confused with blackleg such as, anthrax, the interviewers reviewed with the farmers the clinical signs of blackleg to verify that the respondent had understood the disease correctly. They were then asked questions related to the composition of their herds; occurrence of the disease in their herds in the past 1 year (age, sex and breed of animals affected and died); their practices on treatment and vaccination, cost of treatment/vaccination (whether or not the vaccine given against blackleg was confirmed from the district veterinary clinics). All the data were recorded for subsequent analysis and calculation of production loss attributed to the disease.

### Financial impact assessment

The total cost (C) of a disease is calculated as the sum of production loss (L) (both direct and indirect) and expenditures (E) incurred to control the disease (Otte and Chilonda 2000). Mathematically, C = L + E. The annual financial losses due to blackleg from farmer’s perspective were calculated as the sum of the values of the annual production losses due to mortality and morbidity and the costs for treatment and vaccination. The economic model by Bennett and Ijpelaar (2005), and Kivaria et al. (2007) as cited in Gari et al. (2011) was adopted for the estimation of financial loss attributed to blackleg with slight modification in some of the variables in the equation to best fit to blackleg.

1 $${\text{The}}\;{\text{model:}}\quad {\text{C}} = {\text{Md}} + ({\text{B}} + {\text{M}} + {\text{Wop}}) + {\text{V}} + {\text{T}}$$

where C = the total financial costs, Md is the mortality losses, B = the beef production losses, M = the milk production losses, Wop = the work output losses, V = the vaccination costs (because the cost of vaccine is born by farmers) and T = the treatment costs. Since blackleg is an acute disease, beef loss was considered insignificant and this variable was not included in the model.

$$\begin{aligned} {\text{The}}\;{\text{adapted}}\;{\text{model:}}\quad & {\text{C}} = {\text{Md}} + ({\text{M}} + {\text{Wop}}) + {\text{V}} + {\text{T}} \\ & {\text{With:}}\quad {\text{Md}} = {\text{P}} \times {\text{Qi}} \times {\text{U}}\quad {\text{and}}\quad {\text{T}} = {\text{P}} \times {\text{I}} \times {\text{It}} \times {\text{Utv}} \\ & ({\text{M}} + {\text{Wop}}) = {\text{P}} \times {\text{I}} \times {\text{Q}} \times {\text{U}}\quad {\text{V}} = {\text{P}} \times {\text{Iv}} \times {\text{Utv}} \\ \end{aligned}$$

where, P = Size of population at risk, Qi = proportion of disease losses (mortality rate, work output loss), U = unit value of lost output (USD/L of milk lost, USD/work output lost, USD/unit animal), I = annual cumulative incidence of the disease as a proportion of the affected population, Q = quantity of losses attributed to the disease (litre of milk, lost working days), It = proportion of affected population treated, Utv = cost of treatment/vaccination per animal, Iv = proportion of population vaccinated.

Treatment cost represents the expenses incurred by farmers for medications at district veterinary clinics where farmers bring their clinically sick animals for treatment. Blackleg vaccination is practiced every year in all over the districts to prevent/control outbreaks and farmers incur the cost of vaccine.

The annual cumulative incidence of blackleg in female and male animals and the number of lactating cows and draft oxen during the study period were obtained from the survey data. The percentage of milk production and draught output losses in the study groups was calculated using the formula given in Eq. 2 (Bennett and Ijpelaar 2005; Kivaria et al. 2007).

2 $${\text{Percentage}}\;{\text{of}}\;{\text{production}}\;{\text{losses}}\;(\% ) = ({\text{Q}}/{\text{D}}) \times {\text{I}} \times 100$$

Q = quantity of production lost [milk (L)/lactation, draft output in days]; D = Parameters of the breed types without blackleg (Milk off-take/lactation, annual draft output) and; I = cumulative incidence.

Production loss due to mortality was computed based on the weighted average price, determined for each breed, sex, and age group of animals that had died of blackleg. The expenditures incurred for treatment and cost of vaccination associated with blackleg incidence were recorded from individual herd owners and crosschecked with the cost per unit price of the items in their respective district veterinary clinics. The empirical production loss estimation was based on market prices during the 2011/12 year.

### Partial budget analysis

Partial budget analysis was used to estimate the financial benefit of control interventions. This method is used to assess change in costs and benefits resulting from small changes such as the use of a new approach on livestock farm (Dijkhuizen et al. 1995; Rushton et al. 1999; Rushton 2009). If the benefits exceed the costs, then the change would be advantageous for the system. The most important criterion of whether or not to adopt an approach is determined by the marginal rate of return (MRR) obtained from the change (Legesse et al. 2005; Evans 2008; O’Farrell 2010). MRR is calculated as the net benefit divided by the total cost that varies only by using a new approach.

A planned mass vaccination programs were proposed as an approach to control blackleg. The cattle populations in the four selected districts were the target populations (Table 1). The variables estimated in the financial loss assessment from the study groups (Table 2) were applied for the partial budget analysis of the target population. The animal level prevalence of blackleg in the study area of 9.4 % (95 % CI 4.8–16.2) was used as the parameter of blackleg occurrence in the target population. The farm outputs considered in the model were milk production and draft work output.

**Table 1:** Cattle population by sex and age groups in the study districts. *Source*: Zone ARDO (2011)

Sex, age categories	Name of the districts				
	Kedida Gamela	Damboya	Kacha Bira	Angecha	Sum
Male					39,905
Male calves (<1 year)	1082	447	653	693	2875
Bull (1–3 years)	5119	6327	4909	11,772	28,127
Draft oxen	2489	1589	2902	1923	8903
Female					129,360
Female calves (<1 year)	2383	720	1104	1253	5460
Heifers (1–3 years)	12,528	10,777	11,176	10,908	45,389
Dry cows	18,328	11,942	19,287	13,403	62,960
Lactating cows	7498	1361	4691	2001	15,551
Total	49,427	33,163	44,722	41,953	169,265

Among total cattle population, crossbred animals are 28,833

Among lactating cows, the number of crossbred is 2485

Among draft oxen, the number of crossbred is 2074

**Table 2:** Number of cattle in the study population categorized by sex, age, and breed types based on the questionnaire survey

Description	Local zebu	Crossbred	Sum
Total head of cattle	2631	1077	3708
Male cattle	809	135	944
Female cattle	1822	942	2764
Dry cows	339	276	615
Lactating cow	595	321	916
Oxen (draft)	711	95	806
Bulls/heifers	918	332	55
Calves	68	53	121

The variable costs were the cost of planned mass vaccination with the assumption that farmers would pay the cost of the vaccine. Cattle in Kembata Tambaro zone are managed extensively under open free grazing system with no supplement. Thus, feed savings for diseased animals (which eat less feed) compared to non-diseased animals was not considered in the model. Furthermore, the opportunity labour cost that the herd owner would spend to vaccinate his/her animals was not taken into account because of the relatively cheap labour cost in the study area. The benefit of blackleg control was calculated as the sum of the production output that would be saved from being lost as the result of disease control in the target population and the treatment cost saved.

### Data analysis

Data entry and management was made using Microsoft Excel sheets. Data analysis was made using Statistical Package for Social Science (SPSS 2007 version 16) software. A general linear model was used to compare the occurrence of blackleg between the different breed, sex and age groups. Risk factors associated with blackleg occurrence were identified using logistic regression model. Odds ratio (OR) estimate was used to determine the degree of association between the risk factors and the disease. Financial cost due to blackleg between breed groups were compared using regression model. In all the analyses, confidence levels at 95 % were calculated, and a p < 0.05 was used for statistical significance level.

## Results

### Financial impact of blackleg on milk yield and draft power output

The average daily milk yield and lactation period for crossbreed cows as estimated by discussants in the study districts were 7 L and 240 lactation days (with a range of 180–300 days). Average daily milk yield of 1.85 L per cow and a total of 180 days lactation period per year for local zebu were obtained from the Central Statistical Agency (CSA 2012). The average duration of milk production loss in lactating cows that were infected by and survived blackleg was 11 lactation-days (with a range of 7–15 days) for both local zebu and cross breed cattle. The average duration that blackleg puts its effect on draft power output on oxen affected by the disease was 7 working days (from 3 to 11 days).

### Description of blackleg based on questionnaire surveys

#### Herders’ awareness

Herders’ knowledge on diagnosis and control of blackleg is presented in Table 3. The result indicates that majority (93.3 %) of the respondents heard about blackleg. 49 % of them mentioned clinical signs typical to blackleg as crepitating sound of affected muscle, lameness, swelling of affected area and sudden deaths. About 63.6 % of them explained the disease affect young animals with good body conditions. Most of the respondents (87.8 %) also believed that the disease could be effectively prevented by vaccination.

**Table 3:** Selected variable of the respondents’ knowledge and attitude on cattle blackleg

Variables	Number of respondents	Percentage
Heard about blackleg		
Yes	308	93.3
No	22	6.7
Clinical signs mentioned		
1. Lameness	23	6.9
2. Swelling of affected area	39	11.8
3. Sudden death	13	3.9
4. Crepitating sound	78	23.6
5. Mentioned 1,2, 3 and 4	162	49
6. Others	15	4.5
Mostly affected category mentioned		
Emaciated and old once	82	24.8
Young with good body condition	210	63.6
All animals	39	11.8
Aware that vaccination prevent the disease occurrence		
Yes	290	87.8
No	40	12.2

#### Incidence of blackleg based on the cross sectional survey

Blackleg incidence in the past 1 year indicates that from the total of 330 herds investigated, 165 were affected by at least one case of blackleg. From these herds, 677 animals were affected by the disease from which 137 died. The cumulative incidence and mortality rates for each breed type, sex and age group are shown in Table 4.

**Table 4:** Cumulative incidence of blackleg infection and mortality rates based on breed, sex and age category from the study districts

Description of events	Local zebu		Crossbred		p value
CuI in study groups (%)	n = 471	17.9 (16.5–19.4)	n = 206	19 (16.9–21.6)	0.381
CuI in male animals (%)	n = 108	13.4 (11.2–15.9)	n = 16	11.9 (7.4–18.4)	0.364
CuI in female animals (%)	n = 363	19.9 (18.2–21.9)	n = 190	20 (17.7–22.9)	0.878
CuI in age category	*p* < 0. 05		*p* < 0. 05		
CuI in calves (%)	n = 3	4.4 (1.5–12.2)	n = 9	16.9 (9.2–29.2)	
CuI in bulls/heifers (%)	n = 329	35.8 (32.8–38.9)	n = 107	32.2 (27–37.4)	
CuI in adults (%)	n = 139	8.4 (7.2–9.9)	n = 90	13 (10.7–15.7)	
Mortality rate in affected study groups (%)	n = 94	3.6 (2.9–4.4)	n = 43	3.9 (2.9–5.3)	0.539
Mortality in males (%)	n = 24	2.9 (2–4.4)	n = 6	4.4 (2–9.4)	0.368
Mortality in females (%)	n = 70	3.8 (3–4.8)	n = 37	3.9 (2.9–5.4)	0.912
Mortality in age category (%)	*p* < 0.05		*p* < 0.05		
Mortality in calves (%)	n = 1	1.5 (0.2–7.9)	n = 1	1.9 (0.3–9.9)	
Mortality in bulls/heifers (%)	n = 66	7.2 (5.7–9)	n = 27	8 (5.7–11.6)	
Mortality in adults (%)	n = 27	1.6 (1.1–2.4)	n = 15	2.2 (1.3–3.6)	

*CuI* cumulative incidence

Cumulative incidence and mortality rate of blackleg in local zebu population were 17.9 % (95 % CI 16.5–19.4) and 3.6 % (95 % CI 2.9–4.4), and cumulative incidence and mortality rate in crossbreds were 19 % (95 % CI 16.9–21.6) and 3.9 % (95 % CI 2.9–5.3) respectively. Comparison of parameters between the two breeds showed no significant difference (p > 0.05). In both local zebu and crossbred population, cumulative incidence and mortality rates were higher in females than in males however; the difference was not statistically significant. Comparison of cumulative incidence and mortality rates between age groups in both breeds showed that young had significantly higher cumulative incidence of blackleg infection and mortality rates than calves and adults (p < 0.05).

Association of potential risk factors for the occurrence of blackleg in between breed, age and sex categories showed that young had higher odds of becoming affected by blackleg than calves and adults [OR = 4.5 (95 % CI 3.8–5.4)] which was statistically significant (p < 0.05). However, there is no significant difference in odds of blackleg infection between local zebu and crossbred animals and also the sex groups of either breed.

### Overall financial cost of blackleg based on questionnaire surveys

The average milk production loss in lactating cow that survived blackleg infection was 20.4 L in local zebu and 77 L in crossbred cattle. The percentage milk loss is 1.2 % (95 % CI 0.24–5.8) in local zebu and 0.92 % (95 % CI 0.15–5.3) in crossbreds. Draft oxen are estimated to work an average of 2 months or 60 working days per year in Ethiopia (Tegegne 1997). The percentage work output lost in draft oxen that survived blackleg infection was therefore 1.56 % (95 % CI 0.37–6.3) for local zebu and 1.39 % (95 % CI 0.3–6.07) for cross breeds.

The costs of annual mortality, morbidity, treatment and vaccination in affected groups are presented in Table 5 and market prices of products and services in Table 6. The overall annual financial costs incurred due to blackleg per head was respectively USD 9.8 (95 % CI 6.7–14.4) and USD 16 (95 % CI 10–24.4) in local zebu and crossbred infected herds.

**Table 5:** Estimated annual financial costs of blackleg in the investigated herds for both local zebu and crossbred cattle (in ‘000 USD)

Variables for financial costs	Local zebu cattle		Crossbred cattle	
	Av. Prodn losses (Q)	Value (USD)	Av. Prodn losses (Q)	Value (USD)
1. Estimated milk losses	2377.6 L	1.617	4961.4	3.374
2. Annual mortality losses	94 (3.57 %)	21.32	43 (3.99 %)	13.416
3. Total work output losses	665.5 days	2.642	79 days	0.314
4. Total treated animals and costs	121 animals	0.206	89 animals	0.169
5. Vaccination coverage and costs	27 %	0.028	22 %	0.237
Total costs in zebu cattle		25.813		17.51
Annual cost per head for zebu		0.0098		0.0163
Grand total (local zebu + crossbred)		43.323		

*Av. Prodn losses* average production losses

**Table 6:** Estimated local market prices of various items in 2011/12 year (in USD)

Parameters estimated (USD)	Local zebu cattle	Crossbred cattle
1. Average weighted cattle value	227	312
2. Traction value of an ox per day	3.97	3.97
3. Draft power value/ox/year	238	238
4. Treatment cost/unit	1.7	1.9
5. Vaccination cost/unit	0.04	0.07
6. Milk price/L	0.68	0.68

### Partial budget analysis: financial benefit of blackleg control

The annual milk production increase calculated as a net benefit was estimated to be 0.58 and 0.43 % of the total milk off-take per lactation for zebu and crossbred dairy cattle respectively. The increase in draft power output in terms of working days both in local zebu and crossbreds was estimated to be 1.09 % of the total draft work output per year. The control intervention was assumed to save the cost of treating clinical cases of blackleg which would be of benefit to the farmer.

The MRR gained from the vaccination was calculated to be 9 (900 %) and the net benefit per head was estimated to be USD 0.4 for local zebu and USD 0.8 for crossbreds respectively (Table 7). An annual vaccination to control blackleg would reduce the financial costs due to the disease by 3.4 % per head in local zebu and 6.9 % in crossbreds.

**Table 7:** Financial benefit of blackleg control through planned vaccination in four districts using partial budgeting model (in ‘000 USD)

No.	Parameters	Breed category		Sum
		Local zebu	Crossbreds	
I	New costs			
	Vaccination cost	5.62	2.02	
II	Revenue forgone			
	Opportunity labour cost	0	0	
	Sub-total costs (I + II)	5.62	2.02	7.64
III	New revenue			
	1. Milk production increase	17.16	12.2	
	2. Draft power output increase	17.73	5.38	
IV	Cost saved			
	Treatment cost	22.44	5.15	
	Sub-total benefit (III + IV)	57.33	22.73	80.06
	Net benefit per head	0.0004	0.0008	
	Percentage of financial benefit from the control per head	3.4	6.9	

## Discussion

Investigation of blackleg in the present study and estimation of costs attributable to the disease revealed considerable financial losses to livelihoods of cattle raisers in Kembata Tambaro zone. However, one constraint researchers faced during disease investigation was that herd health and productivity records are not maintained by herd owners in the study districts. To overcome this, we selected districts which recently had experienced blackleg outbreak and individually interviewed farmers as to how the disease had affected their herds in the previous year. Also to improve the validity of information given by the discussants/respondents and to balance the potential recall bias common to every questionnaire survey, responses were crosschecked and multiple sources of information were considered.

Financial losses estimations on milk yield and draft power output due to blackleg occurrence was comparable with the speculations of veterinary literatures indicating equivalent effect of the disease in cattle productivity (Merck 2005; Radostits et al. 2006; Blood and Radostits 1995). Thus, discussants of the study districts were knowledgeable in identifying livestock problems and able to present, share, and analyze their knowledge to find appropriate solutions which is in line with discussants from different parts of Ethiopia (Eshetu 2003; Rufael et al. 2008; Tadesse 2003; Bereket et al. 2013).

Herders’ knowledge in describing blackleg and their ability of recognizing its effect indicate their expertise in identifying livestock health problems in their localities. They considered clinical signs of blackleg consistently with the descriptions of the disease signs in the standard veterinary text books and literatures (Merck 2005; Sarah and Wilson 2013; Radostits et al. 2006; Blood and Radostits 1995). Majority of them also mentioned vaccination as the only means of preventing blackleg occurrence in their herds; which is also in line with standard veterinary literatures recommending annual vaccination of cattle between 6 months and 2 years of age prior to the anticipated danger period to prevent blackleg occurrence in areas where the disease is enzootic (Merck 2005; Radostits et al. 2006; Blood and Radostits 1995).

In the present study, incidence of blackleg in crossbred animals was higher than local zebu breeds. However, the difference was not statistically significant (p > 0.05). The higher incidence in crossbreds might be due to the genetic difference in susceptibility to disease. Females of both breeds had higher cumulative incidence than males, which is contrary to the report of Sultana et al. (2008) who reported a higher incidence of the disease in males. The variation might be due to the difference in grazing practices. Females in the study districts were kept extensively grazing in the fields which exposes them to easy access for bacterial spores in comparison to male animals which are harnessed in the yoke for ploughing purpose and kept indoor many of the times. However, further study would be necessary to determine the underpinning reasons for the difference in susceptibility between breed types and sex.

The difference in blackleg incidences and mortality rates among age category in both breeds was statistically significant (p < 0.05).Young were highly susceptible to blackleg when compared to calves and adults. This is in line with previous studies (Merck 2005; Sarah and Wilson 2013; Radostits et al. 2006; Blood and Radostits 1995) which reported severity of the disease in young animals of between 6 months and 2 years of ages. In the present study, incidence of blackleg in calves of both breeds was less when compared to young and adults. This might be due to the feeding characteristics of calves, and the difference in factors favouring bacterial multiplication. Calves are usually kept at home and provided with harvested grass that might have contributed to a decreased exposure to bacterial spores in the field. Moreover, calves in endemic area could have obtained a certain protective passive immunity from their dam (Wikse 1997).

Blackleg causes higher financial losses in crossbred cattle in most production parameters when compared to local zebu breeds. However, the difference was not statistically significant (p = 0.08); suggesting that the disease uniformly exerts its impact on both breed categories. The financial loss had a linear relationship with the incidence of disease in each breed type (the higher incidence of blackleg the higher financial losses were estimated in both breeds).

The reduced work output of draft oxen due to blackleg was an important loss for the mixed crop livestock farming system of the study area which was estimated based on the daily market price of traction services. Morbidity of draught oxen leads to reduced crop production through reduction in the area that can be cultivated and lowered yields due to inefficient land preparation and timing. Moreover, the reduced milk production due to blackleg could have high impact on the daily household income generation source of herders in study area.

The estimations of financial benefit of blackleg control through planned annual vaccinations show that the net benefit per head for crossbred cattle was higher when compared with local zebu breeds. The high MRR of 9 (900 %) in the current analysis could signify that the investment in planned vaccination to control blackleg would result in high benefits to farmers, which would in turn lead to an efficient allocation of resources (Rushton 2009; Legesse et al. 2005; Evans 2008). However, this high MRR percentage clearly reflected that the cost incurred by farmers for the control was only that of the vaccination cost. Thus, the minimum costs farmers incur to vaccinate their animals; they would potentially keep away from costly losses resulting from death, illness, reduced fertility and productivity due to the disease.

Overall, this study is the first in its kind to address financial impact of blackleg at mixed crop-livestock farming community in Ethiopia. The study realized indispensable and policy-relevant findings with regard to the disease occurrence and its impact on small holder cattle herders. However, lack of related studies significantly reserved researchers not to evaluate their findings with the existing disease scenario. In addition, mortality rate of blackleg in our findings is smaller when compared to mortality reports in veterinary literatures, indicating unique feature of the disease in Ethiopia that needs further researches to discover the existing disease circumstances.

Purposively selecting discussants in the present study help ensure accuracy of information generated for cost calculation. However, we acknowledge as limitation of this study with regard to herders ability in identifying livestock health problems as they may not be representative for the study population.

## Conclusions

Blackleg is one of the major bacterial infections of cattle associated with tremendous economic losses to herders in many parts of Ethiopia. The financial cost-benefit analysis of a planned vaccination compared with non-vaccination for a 1 year period shows that livestock producers would substantially benefit from vaccination not only financially but also by securing and maintaining the prosperity of farming, and the safety and availability of food. Vaccination as currently practiced in the study districts did not involve majority of cattle population, indicating significant number of cattle to be at high risk of blackleg infection. The authors therefore recommend that farmers in the study area should improve their vaccination coverage of their herds. The efforts of all relevant stakeholders are vital to implement a planned vaccination program prior to the anticipated outbreak periods in each study district so that the number of animals dying of black will be prevented and thereby reduce the loss.
